# Assessment of beneficial fungal microorganism’s bio-efficacy in stimulating morphological and physiological parameters of *Allium cepa* plants grown in soil amended with fish wastes

**DOI:** 10.1186/s12870-022-03965-3

**Published:** 2022-12-29

**Authors:** Reda E. Abdelhameed, Rabab A. Metwally

**Affiliations:** Botany and Microbiology Department, Faculty of Science, Zagazig University, Zagazig, 44519 Egypt

**Keywords:** Arbuscular mycorrhizal fungi, *Allium cepa*, Carbohydrates, Chitinase activity, Fish waste, Glomalin

## Abstract

**Background:**

The increase in the human consumption of fish results in the production of organic fish wastes (FW). For enhanced soil fertility and plant growth at a lower cost and without the negative impacts of chemical fertilizers, these wastes could be employed as a valuable organic fertilizer. To determine the synergistic bio-efficacy of *Trichoderma* sp. and arbuscular mycorrhizal (AM) fungi in stimulating the morphological and physiological characteristics of FW-fertilized *Alium cepa*, as well as to investigate their involvement in boosting soil fertility, the current study was carried out. Overall, eight treatments were applied as follows: AM, *Trichoderma* sp., AM + *Trichoderma* sp., FW, AM + FW, *Trichoderma* sp. + FW, AM + *Trichoderma* sp. + FW, and control. Growth and physiological assessments of onion plants were taken after 8 weeks from FW application.

**Results:**

Our results showed that FW application combined with AM fungi and *Trichoderma* sp. inoculations increased aggregate stability of the soil (glomalin content) and soil chitinase activity. Moreover, using the bio-inoculations along with FW amendments significantly (*p* < 0.05) improved the photosynthetic pigments, protein, carbohydrates, and nutrients content of onion plants. It's interesting to note that the triple interaction of AM + *Trichoderma* sp. + FW led to the greatest increase in plant height, root length, number of leaves, and leaf area as well as total fresh and dry weights of shoots and roots. Besides, AM fungal colonization was at its highest percentage with *Trichoderma* sp. inoculation, although this percentage decreased with FW addition.

**Conclusion:**

We concluded that the combined treatments of AM fungi and *Trichoderma* sp. along with FW application to the soil can be proposed as a successful strategy for plant performance in nutrient-deficient soils as both fungal inoculants are capable of degrading these wastes and converting them into manure suitable for farming so plants can uptake the minerals effortlessly.

**Supplementary Information:**

The online version contains supplementary material available at 10.1186/s12870-022-03965-3.

## Background

The most broadly used agronomic practice is the application of chemical fertilizers, which significantly contributes to increasing output as well as crop yield. However, continuous and excessive use of these fertilizers results in increasing soil acidity and quick decay of soil besides groundwater, other than an increase in crop vulnerability to pests and diseases and the disruption of the natural ecosystem [1, 2]. Because of the developing awareness of environmental preservation and stability [3], organic fertilizers have become a desirable alternative to chemical fertilizers. These organic fertilizers are helpful in low-soil-health circumstances, such as malnutrition, scarce water-holding capacity, excessive nutrient leaching, and poor root development [4–6]. Symbiosis with helpful fungi like arbuscular mycorrhizal (AM) fungi and *Trichoderma* has been predicted as one of the mechanisms of plant tolerance, stress avoidance, and reduction of chemicals used in agriculture. This will help to improve the quality of these soils and plant species' ability to resist this harsh environment.

Onion (*Allium cepa* L.), being a bulbous vegetable, responds well to organic fertilizers addition. According to Gadelrab and Elamin [7] and Petrovic and Pokluda [8], organic fertilizers were more effective in obtaining a greater yield and quality of onions; these fertilizers improved the chemical and physical quality of onions. Fertilizers based on organic matter mixed with plant growth-promoting microorganisms, e.g. AM fungi, *Trichoderma* spp. and plant growth-promoting bacteria such as *Azosprillum*, *Pseudomonas* and *Azotobacter* are the most widely used bio additives [9].

Efficiently, these beneficial fungi can mobilize nutrients in the soil from unavailable to available forms through biological processes [10], allowing the microorganisms to colonize the rhizosphere together with the plants and consequently increase the supply or availability of nutrients to the host plants, along with increasing soil fertility and crop production in sustainable farming [11]. On the other hand, the addition of organic amendments to the soil will reverse soil property degradation. Organic amendments’ beneficial effects include increased plant nutrient availability, augmented humus content and thereby improved water–holding capacity, better soil structure, besides increased microbiology activity [12]. Also, these organic amendments can improve the soil's physical and chemical characteristics, enabling marginal soils to become more productive [13]. In this respect, the application of organic amendments to the soil before AM fungal inoculation has been suggested [14]. Among different organic amendments, fish waste (FW), is capable of promoting plant growth and augmenting the microorganism activity existing in the soil [5, 15]. Jayasundara et al. [16] stated that liquid fertilizer of FW mixed with *Tithonia diversifolia* had a significant growth enhancement of *Abelmoschus esculentus*. Moreover, fermented liquid FW showed significantly higher growth performance and significantly increased the leaf area of *Solanum melongena* [17].

The enhanced growth of plants grown on FW may be attributed to their abundance of chitin, which is nature's second most prevalent polysaccharide after cellulose. Chitin is a polymer of N-acetylglucosamine and is tightly associated with proteins, lipids, pigments, calcium carbonate and extra inorganic compounds [18]. It has a high N content of 6.1%–8.3%, which is a comparable level to other organic fertilizers thus used as N and energy sources by plants and microbes. In addition to providing N, the exoskeletons of crustaceans are also rich in Ca minerals [19].

The use of FW in conjunction with beneficial microbes to discharge nutrients to crops for increased sustainable production has previously been reported [5, 20], as these microbes are essential in organic matter breakdown and release complex compounds for plant utilization. Also, chitin present in FW can be hydrolyzed by several chitinolytic enzymes produced by bacteria, fungi, and plants. Fungi are engaged in organic matter decomposition and protein transformation into soluble N nutrients, amino acids and ammonium [21]. Ahmad et al. [22] stated that plants can access N from FW through microbial breakdown and release of inorganic forms of N (NH_4_^+^ and NO_3_^−^). Also, Nandhini et al. [20] used *Brevibacillus agri, Bacillus cereus, Bacillus licheniformis* and *Brevibacillus parabrevis* for FW breakdown to release amino acids and nutrients to crops.

AM fungi, belonging to the phylum Glomeromycota, are a foremost constituent of the soil microbiota and undoubtedly exemplify the most vital terrestrial symbiotic microbes [23–25]. Researchers have become interested in these fungi as a result of their capacity to establish close relationships with between 70 and 90% of plant species [26, 27] and improve the photosynthesis, growth attributes, and enzyme activities of the plants [24, 28]. *Trichoderma* species are also endophytic saprophytes because they readily colonize the host plant's root surface or cortex and benefit the host plant's health and growth [29–31].

These soil fungi are great sources of chitinases and could be exploited in agriculture, biotechnology, and medical applications to catabolize chitinous waste [6, 32]. By employing shrimp shellfish debris as a substrate for solid-state cultivation, Rattanakit et al. [33] developed chitinase. The ability to hydrolyze chitin present in FW is a characteristic of fungal genus *Trichoderma* such as *T. virens, T. aureoviride, T. citrinoviride,, T. harzianum* as they are capable of producing various polysaccharide degrading enzymes i.e. exochitinases, chitobiosidases and endochitinases [34–36]. Furthermore, an induction of specific root chitinase enzymes during AM fungal symbiosis of *Glomus mosseae, G. intraradices* and *Gigaspora rosea* has been described in several plants [37]. Moreover, the highest rates of chitinase activity were found in the roots of sorghum and tobacco plants inoculated with AM fungi [6, 37].

Furthermore, AM fungi can contribute to soil aggregate stability directly through the physical effect of their network that are formed around soil particles and indirectly via the hyphal exudation of an iron-containing, heat-stable glycoprotein termed glomalin as an aggregate binding agent [38, 39]. Glomalin, a widespread AM fungi produced glycoprotein by mycelium and spore walls of AM fungi, is essential for both ecosystem functioning and ecological restoration and plays an important role in structuring soil as in the low nutrient-deficient soils; as the low productivity and fertility of the soil seriously limit plant growth. Besides, glomalin possibly evolved on the fungal hyphae as a defensive coating to avoid the loss of water and nutrients before reaching the plant host and to protect the hyphae from decay. In addition, glomalin helps to stabilize soil aggregates through the creation of a polymer-like protective lattice on the aggregate surface [40–42]. Medina and Azcón [12] reported that the organic amendment applications improved the soil quality of degraded soils and the plant's ability to resist these conditions. As a result, the goal of this research was to evaluate the utility of fungal inoculums like AM fungi along with *Trichoderma* sp. in improving the quality of soils treated with FW and their enhancing capacity for the growth performance and physiological processes of *Allium cepa* plants. Moreover, this study was conducted to determine whether the addition of FW to the soil can support the bio-fertilizer (AM fungi and *Trichoderma* sp.) in enhancing the nutrient contents of onion plants.

## Results and discussion

### Identification of *Trichoderma* sp

In this study, the morphological identification was confirmed by molecular analysis of ITS rDNA sequence (18S–28S rRNA) (Cai and Druzhinina [43] and Druzhinina, [44]) flanking ITS1 (5.8S rRNA), and ITS2 using ITS1/ITS4 primers as *Trichoderma* sp. The retrieved ITS sequence was deposited in NCBI GenBank under the accession No. ON479613. The sequence data were aligned for their similarity with the other related strains with BLAST program. Molecular evolutionary genetic analysis (MEGA version 7) software was used for phylogenetic analysis [45]. Phylogenetic tree as shown in Supplementary file (Fig. S1) was made using neighbor-joining method with 1000 bootstrap replicates based on ITS gene sequences. The closets homologous to the sequences were selected, and multiple sequence alignments were carried out using the ClustalW program in the MEGA7 software. The evolutionary distances were computed using the maximum composite likelihood method.

### Mycorrhizal colonization

**Fig. 1:**
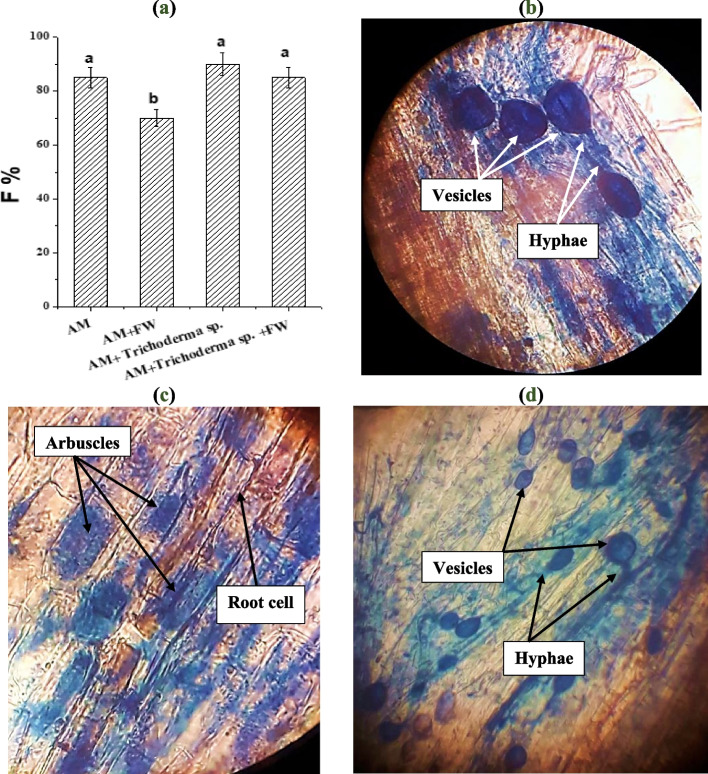
**a** Effect of *Trichoderma* sp. inoculation and FW application on AM fungal colonization (F%) of onion plant roots. **b**, **c** and **d** Photomicrographs of the stained roots of onion (10X) showed structural colonization of AM fungi showed hyphae, arbuscules and vesicles in root cells

According to Sutton [46], there are three phases to AM fungal colonization: (1) a lag phase during which spore germination, germ tube growth, and initial penetration occur; (2) a rapid growth phase during which the fungus spreads throughout the roots and develops external mycelia; and (3) a stable phase during which the ratio of colonized roots to non-colonized ones stays relatively constant. Different AM fungal species inoculating onion plants resulted in a significant increase in AM fungal root colonization (F%) (Fig. 1a), which is shown in Fig. 1b, c, and d as various colonization forms [intraradical (IH), vesicles (V), and arbuscules (Arb)], whereas colonization was not seen in onion roots that had not been inoculated with AM. Although, Ellyzatul et al. [15] found that FW application increased the activity of microorganisms present in the soil. Besides, Johansson et al*.* [47] reported that organic fertilizers addition favors bacterial communities that encourage spore germination and increase the rate of AM fungal colonization. Our findings indicated that the high availability of macro and micronutrients added to the soil through FW, particularly the amount of P [48] that increases the plant photosynthetic rate and increases the amount of sucrose in the plant, caused a decrease in AMF root colonization compared with AM plants [49]. Similar results were reported [6]. Also, Sousa et al. [50] observed a decrease in AM fungal root colonization percentage in cowpea plant roots with manure and gliricidia applications. However, our results showed a significant increase in F% with *Trichoderma* sp. application in comparison to FW. These findings are in agreement with Metwally [28] who stated synergistic influence of *T. viride* on AM fungal colonization in onion roots because the effects of the soluble and volatile exudates produced by *Trichoderma* are complicated [31, 51]. Also, *Trichoderma* released volatile compounds that stimulate the development of AM fungal auxiliary cells [30, 52].

### Plant growth performance

With the application of FW, there was an improvement in the onion growth traits (Tfwt, TDwt, plant height, root length, leaf number, bulb and neck diameter), although this increase was not significant (Table 1). This result was not consistent with Ranasinghe [3] and Ellyzatul et al. [15] who reported that FW application significantly increased the leaf number, leaf area, shoot length and fruit weight of *Albemonchus esculentus* and cucumber plants. This enhancement may be attributed to the improvement in soil structure and elevation in the plant growth regulators and levels of plant nutrients as FW is a rich source of proteins, carbohydrates and minerals like P, Zn, N, Ca, K, Mg, and Fe which increased root growth and leaf area of onion plants [53]. As shown in our previous recorded data [5] for macro and micronutrients analysis of FW, results showed that FW is rich in N (7.01%), P (12,201 µg/g), K (1573 µg/g), Ca (89,762.5 µg/g), Mg (189 µg/g), Fe (266.81 µg/g) and Zn (94.37 µg/g). Also, the increase in onion growth attributes due to FW application could be a result of increased phosphatase and chitinase activity [54].

**Table 1:** Effect of AM fungi, *Trichoderma* sp. inoculations and FW application on different growth traits of onion plants

Treatments	Plant Height (cm)	Root length (cm)	Leaf area (cm^2^)	Bulb diameter (cm)	Neck diameter (cm/plant)	Leaves number (No/plant)	Total Fresh weight (TFwt)	Total Dry weight (TDwt)
Control	e54 ± 2.47	31.3 ± 1.43d	.84 ± 2.1e54	4.1 ± 0.187d	3.6 ± 0.165ef	6 ± 0.27b	25.84 ± 1.184e	1.9512 ± 0.089d
AM	62.6 ± 2.87bc	39 ± 1.78ab	79.24 ± 3.63b	4.5 ± 0.206 cd	4.3 ± 0.197bc	6 ± 0.26b	32.98 ± 1.511c	2.598 ± 0.119c
FW	57.2 ± 2.62de	34 ± 1.56 cd	60.02 ± 2.75d	4.4 ± 0.202 cd	3.9 ± 0.178de	6 ± 0.28b	27.44 ± 1.257de	2.0663 ± 0.095d
AM + FW	66.6 ± 3.05ab	40 ± 1.83a	83.47 ± 3.8ab	4.6 ± 0.21c	4.5 ± 0.206b	6 ± 0.27b	35.88 ± 1.644b	2.907 ± 0.133b
*Trichoderma* sp*.*	57.1 ± 2.62de	34.2 ± 1.57 cd	58.8 ± 2.69de	4.4 ± 0.202 cd	3.4 ± 0.156f	6 ± 0.27b	29.74 ± 1.36d	2.153 ± 0.099d
AM + *Trichoderma* sp.	63.4 ± 2.91abc	40 ± 1.83a	70.6 ± 3.23c	5.4 ± 0.247b	4.4 ± 0.201bc	6 ± 0.27b	35.27 ± 1.62bc	2.8501 ± 0.131b
*Trichoderma* sp. + FW	60.1 ± 2.75 cd	36.2 ± 1.65bc	66.3 ± 3.03c	4.7 ± 0.215c	4.1 ± 0.187 cd	6 ± 0.31b	32.57 ± 1.49c	2.5458 ± 0.117c
AM + *Trichoderma* sp. + FW	67.9 ± 3.11a	42 ± 1.92a	88.5 ± 4.05a	6.4 ± 0.293a	4.9 ± 0.224a	8 ± 0.36a	39.4 ± 1.81a	3.271 ± 0.149a

Values reported are means of 5 replicates with standard deviation. Different letters in each column indicate significant differences among treatments using a one-way ANOVA followed by the Duncan’s multiple range test (*p* < 0.05). Three-way ANOVA results are shown in Table 2*Control: onion plants non-inoculated with AM fungi or *Trichoderma* sp.; AM: onion plants inoculated with AM fungi; FW: onion roots treated with fish waste; AM + FW: onion roots treated with AM fungi and fish waste; *Trichoderma* sp.: onion plants inoculated with *Trichoderma* sp.; AM + *Trichoderma* sp.: onion plants dually inoculated with AM fungi and *Trichoderma* sp.*; Trichoderma* sp. + FW: onion roots treated with *Trichoderma* sp. and fish waste and AM + *Trichoderma* sp. + FW: onion roots dually inoculated with AM fungi and *Trichoderma* sp.with fish waste

**Fig. 2:**
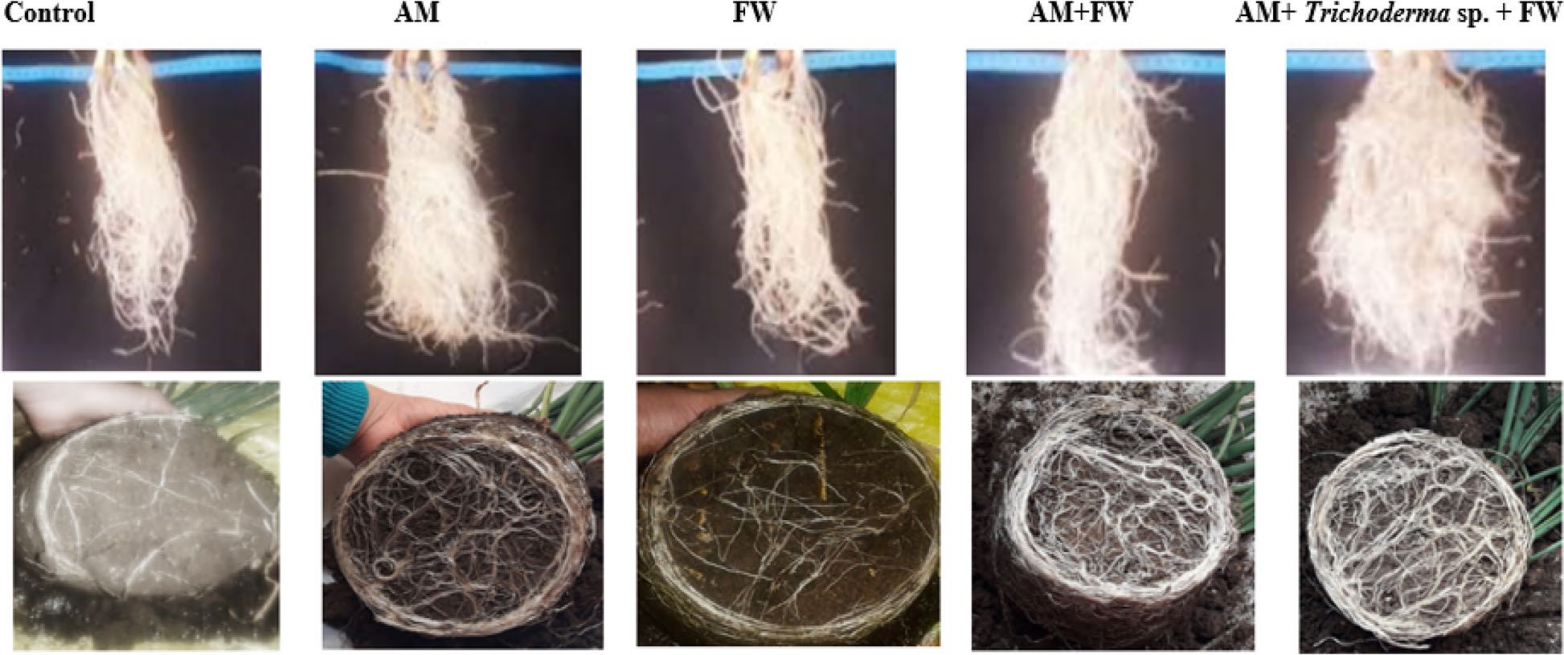
Morphology of onion roots after 8 weeks of growth in soil with AM fungi, *Trichoderma* sp. inoculations and FW application. Control: onion roots non-inoculated with AM fungi or *Trichoderma* sp.; AM: onion roots inoculated with AM fungi; FW: onion roots treated with fish waste; AM + FW: onion roots treated with AM fungi and fish waste and AM + *Trichoderma* sp. + FW: onion roots dually inoculated with AM fungi and *Trichoderma* sp. with fish waste

A noteworthy finding was that an additional boost in onion growth parameters like bulb and neck diameter with their triple interaction (AM fungi, *Trichoderma* sp. and FW application) was appeared through results of three-way ANOVA (Table 1, Fig. 2) compared to when each fungus was administered separately. These results support Abdelhameed and Metwally's hypothesis [55] that extraradical mycorrhizal hyphae are involved in the mycorrhiza-mediated enhancement of plant nutrition. Moreover, statistical analysis indicated that AM fungal inoculation with FW had the highest statistical significance on both TFwt and TDwt of onion plants, followed by *Trichoderma* sp. According to Metwally and Al-Amri [30], AM fungus and *T. viride* dual inoculation improved Fwt and Dwt, plant height, and root length of *Allium cepa* plants in comparison to the control plant, findings that are consistent with our investigation. Compared to control plants, onion plants dual-inoculated with AM fungi and *Trichoderma* sp. in FW applied soils improved by 25.7%, 35.5% and 36.1% in plant height, root length, and neck diameter, respectively. The synergistic effect of *Trichoderma* sp. with AM fungi, which made P, N, K and micronutrients like Fe, Zn, Mn, and Cu from FW more soluble and subsequently enhanced their uptake with AM fungi, may support this. The decomposition of the FW was enhanced by the activity of these two types of microorganisms (AM and *Trichoderma* sp.) in the soil, which also improved the transfer of organic matter and other nutrients to the soil, supplying more nutrition for the plant's growth [21].

### Photosynthetic pigments contents

**Fig. 3:**
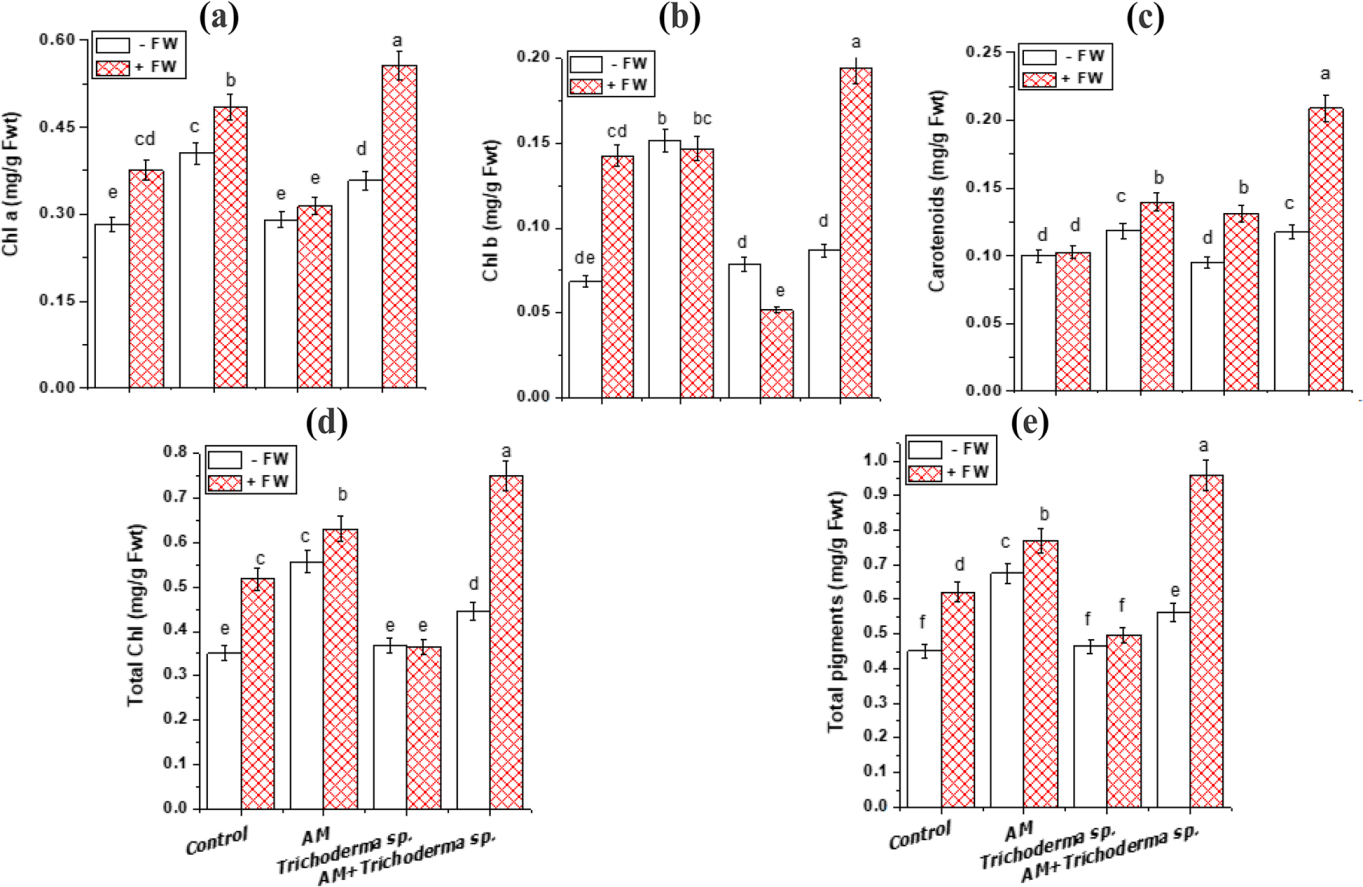
Effect of AM fungi, *Trichoderma* sp. inoculations and FW application on pigment fractions (mg/g Fwt) of onion leaves; **a** Chl a, **b** Chl b, **c** Carotenoids, **d** Total Chl and (**e**) Total pigments. Values reported in figures are means of 5 replicates with standard deviation. Different letters on the bars indicate significant differences among treatments using a one-way ANOVA followed by the Duncan’s multiple range test (*p* < 0.05). Three-way ANOVA results are shown in Table 2. *Control: onion plants non-inoculated with AM fungi or *Trichoderma* sp.; AM: onion plants inoculated with AM fungi; *Trichoderma* sp.: onion plants inoculated with *Trichoderma* sp.; AM + *Trichoderma* sp.: onion plants dually inoculated with AM fungi and *Trichoderma* sp

**Fig. 4:**
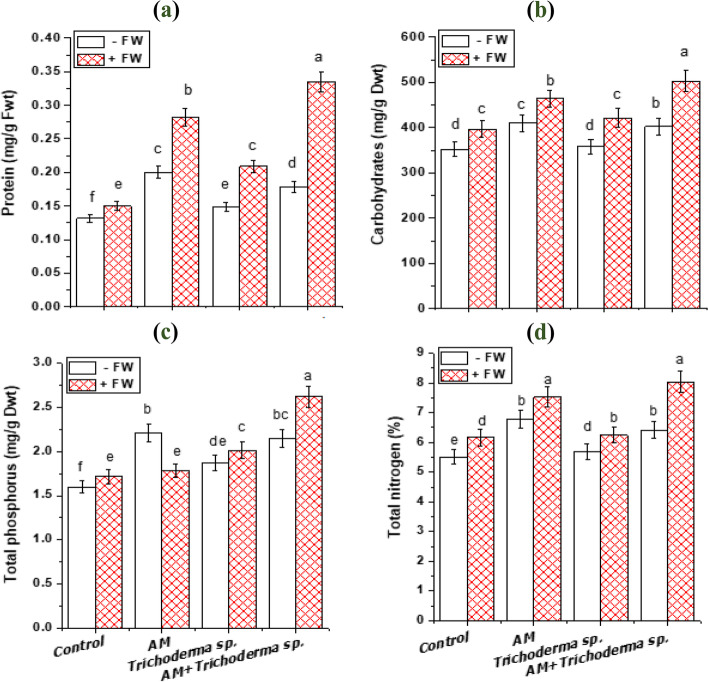
Effect of AM fungi, *Trichoderma* sp. inoculations and FW application on: (a) protein content (mg/g Fwt root); (b) carbohydrates content (mg/g Dwt shoot); (c) total phosphorus (mg/g Dwt) and (d) total nitrogen (%) of onion plants. Values reported in figures are means of 5 replicates with standard deviation. Different letters on the bars indicate significant differences among treatments using a one-way ANOVA followed by the Duncan’s multiple range test (*p* < 0.05). Three-way ANOVA results are shown in Table 2 *Control: onion plants non-inoculated with AM fungi or *Trichoderma* sp.; AM: onion plants inoculated with AM fungi; *Trichoderma* sp.: onion plants inoculated with *Trichoderma* sp.; AM + *Trichoderma* sp.: onion plants dually inoculated with AM fungi and *Trichoderma* sp

Precision agriculture has been reported to place a special focus on the importance of leaf chlorophyll concentration as a good indication of photosynthesis and plant productivity [21]. Our results revealed that FW, AM fungi and *Trichoderma* sp. single inoculation increased the total chlorophyll contents of onion plants by 47.5%, 58.6% and 5.1%; respectively compared with the control (Fig. 3d). These results are matching with the previous studies on cowpea and fenugreek plants [55, 56]. Besides, Begum et al. [57] stated that the increase in photosynthetic activities of plants is directly associated with the improved growth frequency of AM fungal inoculation that is directly linked to the N and P uptake (Fig. 4), which move towards roots and stimulate the growth parameters (Fig. 2). Moreover, AM plants sequester more Zn through the means of extraradical mycorrhizal hyphal structure and improve Mg uptake (which are critical constituents necessary for photosynthesis), leading to an increase in chlorophyll concentrations, consequently increasing photosynthate production, stomatal conductance, transpiration rate and carbon assimilation as well as plant progress [24]. Additionally, the rise in chlorophyll content (Fig. 3a, b and d) with FW application is consistent with Liu et al. [21] and Dalorima et al. [58] in pineapple and watermelon. Similarly, Khandaker et al. [59] reported that fermented fish extract application increased the chlorophyll content of chilli plants. The stimulatory effect of FW on photosynthesis might be due to its richness with Zn and Fe which are required for plant metabolism and chlorophyll biosynthesis. Conversely, Ellyzatul et al. [15] reported that FW application did not affect chlorophyll a, b and carotenoid content of cucumber leaf.

Noticeably, the highest value of carotenoids was shown with AM, *Trichoderma* sp. and FW triple interaction (0.209 ± 0.0095 mg/g Fwt) followed by AM and FW dual treatment (0.139 ± 0.0064 mg/g Fwt), as compared with AM and *Trichoderma* sp. single inoculation (0.118 ± 0.0054 and 0.095 ± 0.0043 mg/g Fwt); respectively as shown in Fig. 3c. The maximum amplification in onion pigments content (Fig. 3e) dually treated with AM fungi and *Trichoderma* sp. and applied with FW was in line with the results of Metwally and Al-Amri [30]. These are consistent with Cruz et al. [60] who reported that soil enrichment with organic matter increased the carotenoids content in lettuce leaves.

### Total soluble carbohydrates content

The contents of soluble sugar are related to the plant's growth potential and stress-resistance ability [21]. Based on the results of three-way ANOVA, there was a significant increase in total soluble carbohydrates contents with AM, *Trichoderma* sp. and FW interactions (Table 2). As a representative in Fig. (4), total soluble carbohydrates in onion plant leaves were increased by 12.9, 16.5 and 1.9% with FW additions, AM and *Trichoderma* sp. application applied singly which was consistent with the previous results of Metwally and Abdelhameed [27] and Metwally [28] that attributed this increase to the enhancement in photosynthesis that allowed higher sugars distribution from shoots to roots and hydrolysis of starch to soluble sugar in AM plants. Furthermore, the augmentation in soluble carbohydrates as a result of *Trichoderma* sp. application may be a result of the increase in total chlorophyll content as our results showed (Fig. 3d). This result agreed with Badar and Qureshi [61] findings in the combination of *T. hamatum* and *Vigna mungo*-specific *Rhizobium*. Also, the maximum amplification in soluble carbohydrates content was reported in onion plant leaves dually treated with AM fungi and *Trichoderma* sp. and applied with FW as clearly apparent in Fig. (4). The increase in total soluble carbohydrates content with FW application was in line with Liu et al. [21] who reported that the composted pineapple markedly augmented the soluble sugars contents. This may be attributed to an increase in the amount of organic matter and an improvement in the physical and chemical aspects; composted residue provides a better soil environment and a plentiful source of carbohydrates and N that promote the growth of beneficial microorganisms [62]. Contrary to our results, Nurbaity et al. [13] stated that the interaction between organic matter such as compost and biochar and AM fungi had no significant effects on the protein, lipid and carbohydrate contents of potato tubers.

**Table 2:** Significance levels (**F**-values) of FW, AM fungi, ***Trichoderma* sp.** and their interactions on measured variables based on two-way or three-way ANOVA analysis

Variables	Waste	AM fungi	*Trichoderma* sp.	AM fungi x Waste	*Trichoderma* sp. x Waste	AM fungi x *Trichoderma* sp.	AM fungi x *Trichoderma* sp. x Waste
Plant height	10.275*	48.99*	3.12 ns	15.25*	0.004 ns	0.723 ns	12.25*
Root length	7.63*	82.374*	8.443*	0.372 ns	0.012 ns	0.568 ns	0.372 ns
Leaf area	44.9*	241.57*	1.97 ns	2.619 ns	8.109*	7.62*	5.25*
Bulb diameter	21.83*	82.23*	82.2*	1.88 ns	6.11*	33.31*	6.12*
Neck diameter	29.77*	99.02*	2.58 ns	0.927 ns	5.1*	2.58 ns	0.103 ns
TFwt	21.97*	130.6*	36.851*	1.12 ns	1.74 ns	1.01 ns	20.21*
TDwt	54.8*	254.2*	60.088*	4.938*	0.722 ns	9.25*	0.055 ns
Chl a	178.3*	337.4*	0.906 ns	2.97 ns	29.57*	7.15*	41.04*
Chl b	259.7*	651.1*	111.24*	35.68*	1.52 ns	46.6*	527.2*
Carotenoids	237.7*	251.8*	88.9*	56.9*	111.29*	20.76*	13.74*
Total chl	197.9*	406.41*	10.67*	31.19*	2.61 ns	13.58*	108.55*
Total pigments	206.59*	373.7*	0.486 ns	35.95*	11.77*	14.98*	82.43*
Protein	389.3*	492.1*	44.7*	97.4*	52.29*	7.36*	4.06 ns
Carbohydrates	71.95*	28.31*	22.86*	1.59 ns	22.86*	0.001 ns	0.719 ns
Total nitrogen	52.53*	108.29*	0.723 ns	5.52*	2.368 ns	0.059 ns	3.937 ns
Total phosphorus	3.9 ns	107.66*	79.4*	0.169 ns	37.05*	1.66 ns	33.86*
Chitinase	56.3*	234.6*	67.8*	27.49*	0.067 ns	12.14*	19.2*
Easily extractable glomalin	49.648*	139.573*	149.149*	3.453 ns	16.934*	1.761 ns	20.40*
Total extractable glomalin	94.907*	101.767*	14.049*	14.704*	5.720 ns	29.065*	36.603*

Significance levels: *p < 0.05; ns, non-significant effect

### Total soluble protein content

Protein is an essential compound in food, in which its concentration in plants is highly dependent on N availability in the soil at sowing time; N is released during the growing seasons through mineralization of soil organic matter [13]. In general, the tripartite interaction of FW addition, *Trichoderma* sp., and AM fungal inoculation significantly affected the protein content of onion roots (Table 2**)**. Based on Fig. (4), in comparison to plants that weren't treated, FW amendments caused an increase in the amount of onion root protein in AM and *Trichoderma* sp. inoculated plants. When onion roots were dual inoculated with AM and *Trichoderma* sp. and supplemented with FW, its highest level was detected **(**0.335 ± 0.015 mg/g Fwt) compared to control ones. These are in accordance with results that reported in sorghum plants inoculated with *Glomus intraradices* and amended with FW which exhibited an increase in total soluble protein [6]. Where, proteins can be either covalently linked within the AM fungal cell wall or they can non-covalently associate with the wall of the plant roots [57]. Also, the vital role of AM fungi in protein stimulation may be attributed to AM fungi facilitating certain plant genes activation and nutrient accumulation [23, 27, 63, 64]. In addition, *Trichoderma* sp. significantly increased the protein content of the roots in the majority of non-AM and AM plants treated with or without FW. This is consistent with our latest research with *Allium cepa* plants that were dual-inoculated with AM fungus and *Trichoderma* sp. and treated with FW [5]. In a similar manner, the seed protein of *Vigna sinensis* was also shown to be increasingly affected by FW, *Pseudomonas* bacteria, and their interactions [65]. This increase may be a result of increased biological N fixation, nitrate uptake, and formation of nitrogen oxide converted to the enzyme L-arginine, which is essential for protein biosynthesis [66].

### Chitinase enzyme

As a result of the high cost of chitinase as well as expensive pretreatment operations of the crab shell and shrimp, the synthesis of chitin and its hydrolyzed derivatives, such as acetylglucosamine and chitooligosaccharides from fish industry wastes has been limited [15, 32, 67]. So, using chitinases produced by chitinolytic microbes for effective bioconversion of chitinous waste has become increasingly essential. Microorganisms are regarded as the preferred chitinases source due to their huge abundance in nature and the simple availability of raw materials, resulting in cheaper chitinases manufacturing costs [32].

**Fig. 5:**
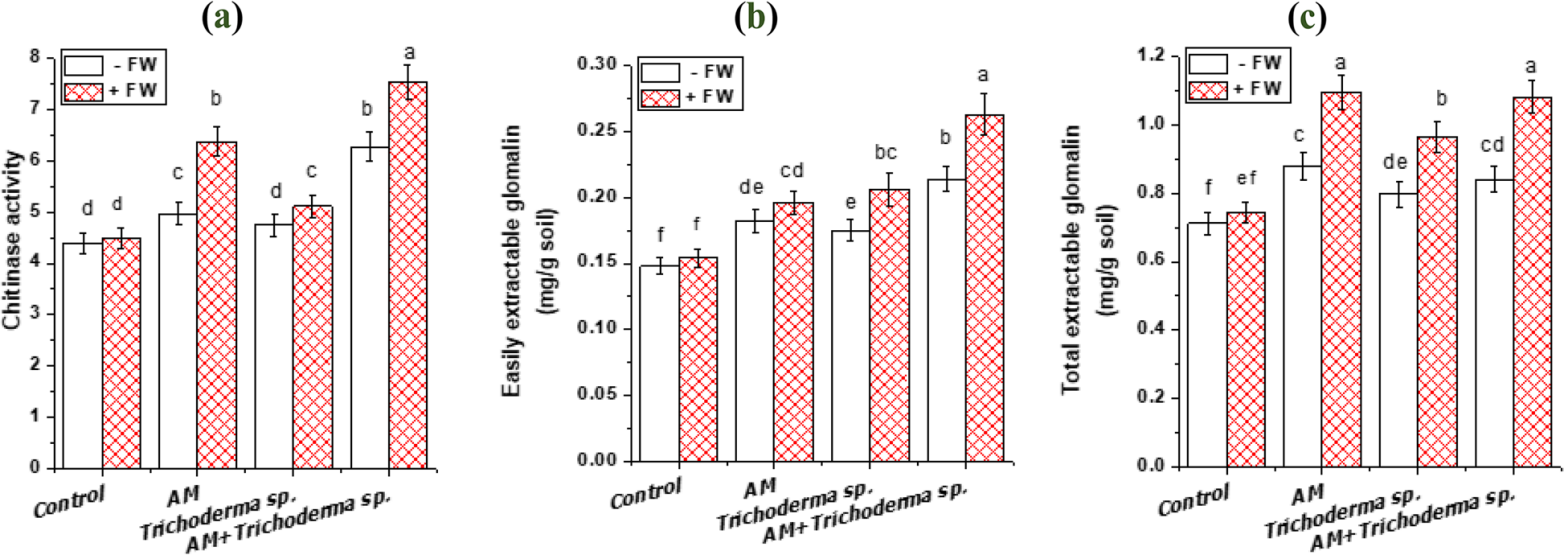
Effect of AM fungi, *Trichoderma* sp. inoculations and FW application on: (**a**) chitinase activity (μmol of N-acetyl-d-glucosamine /g soil) and (**b** and **c**) easily and total extractable glomalin related soil protein (mg /g soil). Values reported in figures are means of 5 replicates with standard deviation. Different letters on the bars indicate significant differences among treatments using a one-way ANOVA followed by the Duncan’s multiple range test (*p* < 0.05). Three-way ANOVA results are shown in **Table** **2**. *Control: onion plants non-inoculated with AM fungi or *Trichoderma* sp.; **AM**: onion plants inoculated with AM fungi; ***Trichoderma*** **sp.**: onion plants inoculated with *Trichoderma* sp.; **AM + *Trichoderma*** **sp.**: onion plants dually inoculated with AM fungi and *Trichoderma* sp.

**Fig. 6:**
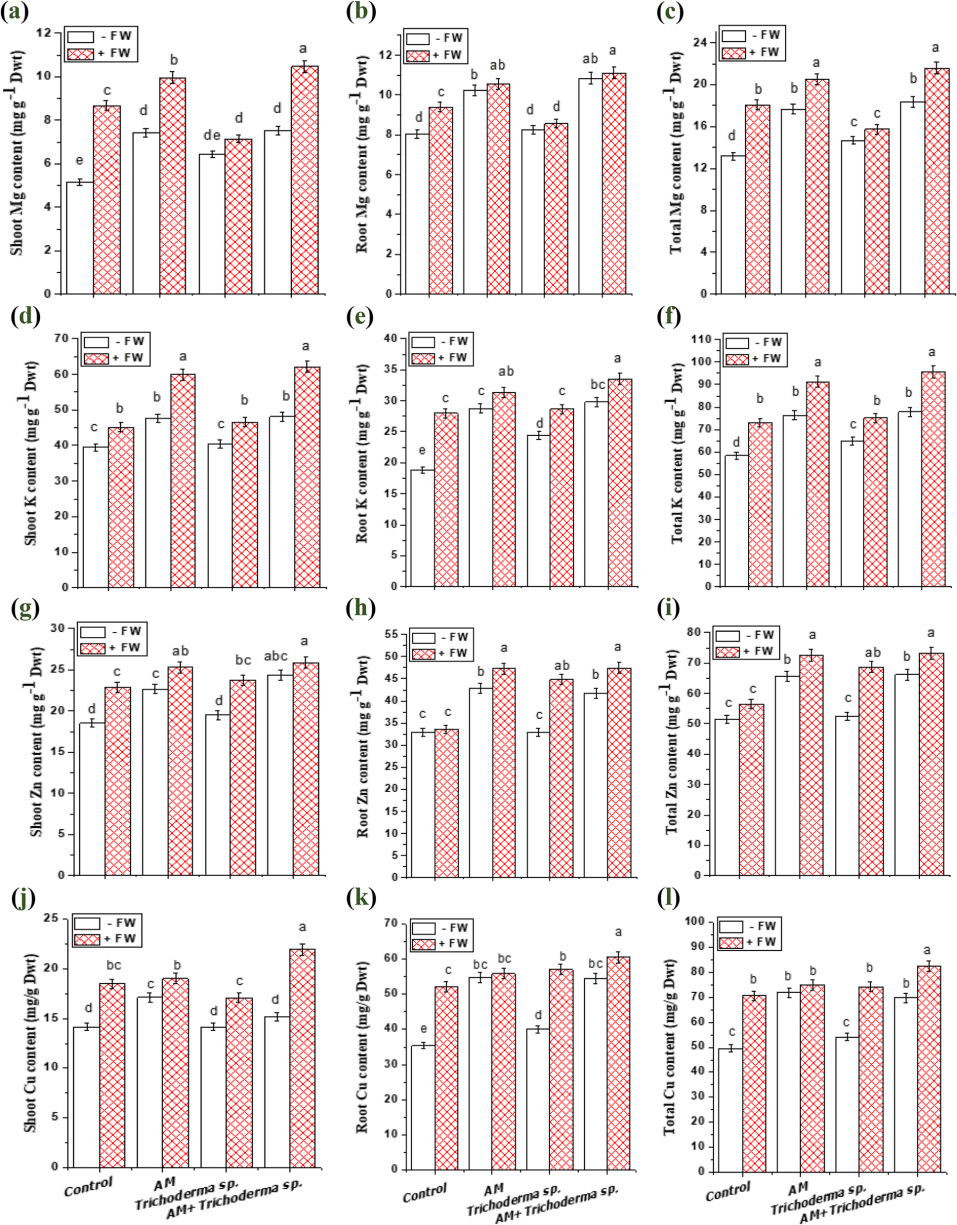
Effect of AM fungi, *Trichoderma* sp. inoculations and FW application on minerals content of onion plants; (**a**, **b** and **c**) for Mg content, (**d**, **e** and **f**) for K content, (**g**, **h** and **i**) for Zn content and (**j**, **k** and **l**) for Cu content. Values reported in figures are means of 5 replicates with standard deviation. Different letters on the bars indicate significant differences among treatments using a one-way ANOVA followed by the Duncan’s multiple range test (*p* < 0.05). *Control: onion plants non-inoculated with AM fungi or *Trichoderma* sp.; **AM**: onion plants inoculated with AM fungi; ***Trichoderma*** **sp.**: onion plants inoculated with *Trichoderma* sp.; **AM + *Trichoderma*** **sp.**: onion plants dually inoculated with AM fungi and *Trichoderma* sp.

The soil chitinase enzyme activity was significantly influenced by the three variables (AM, *Trichoderma* sp. and FW), and AM fungal inoculation had the maximum pronounced effects (Table 2, Fig. 5). Where, soil treated with FW alone or in combination with AM fungi and *Trichoderma* sp. led to higher chitinase activity than control soil (Fig. 6). Highly significant differences in soil chitinase activity were detected between AM and non-AM onion plants grown in soils treated or untreated with FW. Our results are consistent with Brzezinska et al. [32] reports that shrimp waste was used in agriculture as an economical natural N fertilizer, whereas earlier as [68], Brzezinska et al. documented their decomposition by microorganisms.

The significant increase in chitinase activity in soils applied with AM onion plants was in accordance with Pozo et al. [37] who reported that chitinase activity was higher in AM tomato roots, where their stimulation appears as a specific response to AM fungal symbiosis. Also, these results confirmed the previous report of distinguishing three chitin synthase gene proteins during colonization of *Gigaspora margarita* by a reverse transcription-polymerase chain reaction [69]. In this regard, Abdel-Fattah and Mohamedin [6] revealed that AM sorghum plants considerably exceeded non-AM plants in terms of soil chitinase activity in both control and chitin-amended soils. Likewise, the increase in soil chitinase activity with *Trichoderma* sp. application was in line with Haran et al. [70] who stated that *T. harzianum* produces two N-acetylglucosaminidases, four endochitinases, and one chitobiosidase. Also, *T. saturnisporum* produces Chitobiosidase [71].

### Glomalin related soil protein (GRSP)

Glomalin, a glycoprotein at least partially formed by AM fungi, plays an important role in soil structuring. The role of AM fungi in increasing the GRSP content has been demonstrated by several studies [39, 41, 72]. GRSP, which consists of proteins, carbohydrates, aliphatic components and multivalent cations (i.e. Fe and Al), acts as a glue to bind soil particles [38, 39]. Where, cations similar to Fe and Al could encourage compound precipitation and build bridges between particles of clay and organic matter that result in soil aggregation [39, 42].

Figure 6 shows that the concentrations of T-GRSP and EE-GRSP in rhizospheric soil of *Alium cepa* increased with AM, *Trichoderma* sp. and FW application, varying from 1.082 ± 0.049 mg\g for T-GRSP and 0.263 ± 0.036 mg\g for EE-GRSP as opposed to control one (0.712 ± 0.033 and 0.148 ± 0.0068 mg /g soil respectively). Additionally, the EE-GRSP and T-GRSP in soils were significantly affected by the interactions between the three variables, according to ANOVA results (Table 2, Fig. 5). Although there was a decrease in AM fungal colonization with FW application, there was an increase in GRSP with FW. According to Gryndler et al. [73], humic substances, such as fulvic acids that result from the organic fertilizers decomposition, adsorb free cations from the soil solution and may favor the physiological functions of the fungal mycelia (absorption and transport). Also, improving the soil structure contributes to the production of AM fungal mycelia. Additionally, the results shown in Fig. 6 demonstrated that EE-GRSP and T-GRSP were much greater in the soils of AM-inoculated plants than they were in the soils of non-AM-inoculated plants. This was consistent with Cheng et al. [39] who stated that *F. mosseae* considerably increased the contents of EE-GRSP and T-GRSP under well-watered and drought stress conditions. Also, Yang et al. [41] suggested the important role of AM fungi in aggregate stability under heavy metals stress conditions. Sousa et al*.* [50] also observed that when compared to the control, there was a substantial impact of organic fertilization on GRSP content and an increase in the GRSP content was observed in the rhizosphere of maize and cowpea at all fertilization systems. It is probable that the AM fungi augmented the propagules number, which is used as an existence strategy, and favored the increase of glomalin production. Glomalin accumulation in the soil, according to Driver et al. [74] and Meng et al. [75], is due to the decomposition of spores and hyphae and, to a lesser degree, to the passive release or secretion of the hyphae.

### Mineral nutrient analysis

Excessive land use might have a dramatic effect on overall biodiversity, which, as shown by many studies, can, in turn, affect the role of the environment [57]. A prominent characteristic of the AM fungal relationship with plants is the transfer of nutrients [76]. AM fungal inoculation can significantly boost the concentration of different macro-nutrients and micro-nutrients, leading to increased production of photosynthate and thus increased accumulation of biomass [28, 77]. The results concerning the effect of FW additions in the presence and absence of AM fungi and *Trichoderma* sp. application on macronutrients (N, P, K, Ca and Mg) of onion shoots and roots are shown in Fig. (4 and 6). The interactions between FW, AM, and *Trichoderma* sp. had a significant impact on the nutritional contents of the onion shoot and root, according to the ANOVA results. Our results of increased macronutrients in onion tissues coincide with the latest reports by Metwally [28] and Metwally and Al-Amri [30] of increases in leaf area, and N, K, Ca, and P contents, reflecting enhancement in plant growth. The probable explanation is that *Trichoderma* works independently to solubilize inorganic minerals and boost plant nutrient absorption [78]. Also, AM fungi produce fungal structures like arbuscules and extra-radical mycelium, which assist and improve in exchange of inorganic minerals and nutrient uptake, ultimately imparting a considerable vigor to host plants [57]. Similarly, FW added to the control soils caused an increase in the nutrient accumulation in both onion shoot and root tissues, because FW is a rich source of minerals like P, Ca, Mg, K, Zn, Fe as well as N (the main source of soil nutrition besides a well-known mineral fertilizer) [53]. One interesting finding was that FW, AM and *Trichoderma* sp. application results in higher proliferation in onion total P, N and K (64.1, 45.9 and 63.6%) than that of AM (38.7, 23.1 and 30.8%) or *Trichoderma* sp. single inoculated (17.2, 3.5 and 11.1%); respectively.

**Fig. 7:**
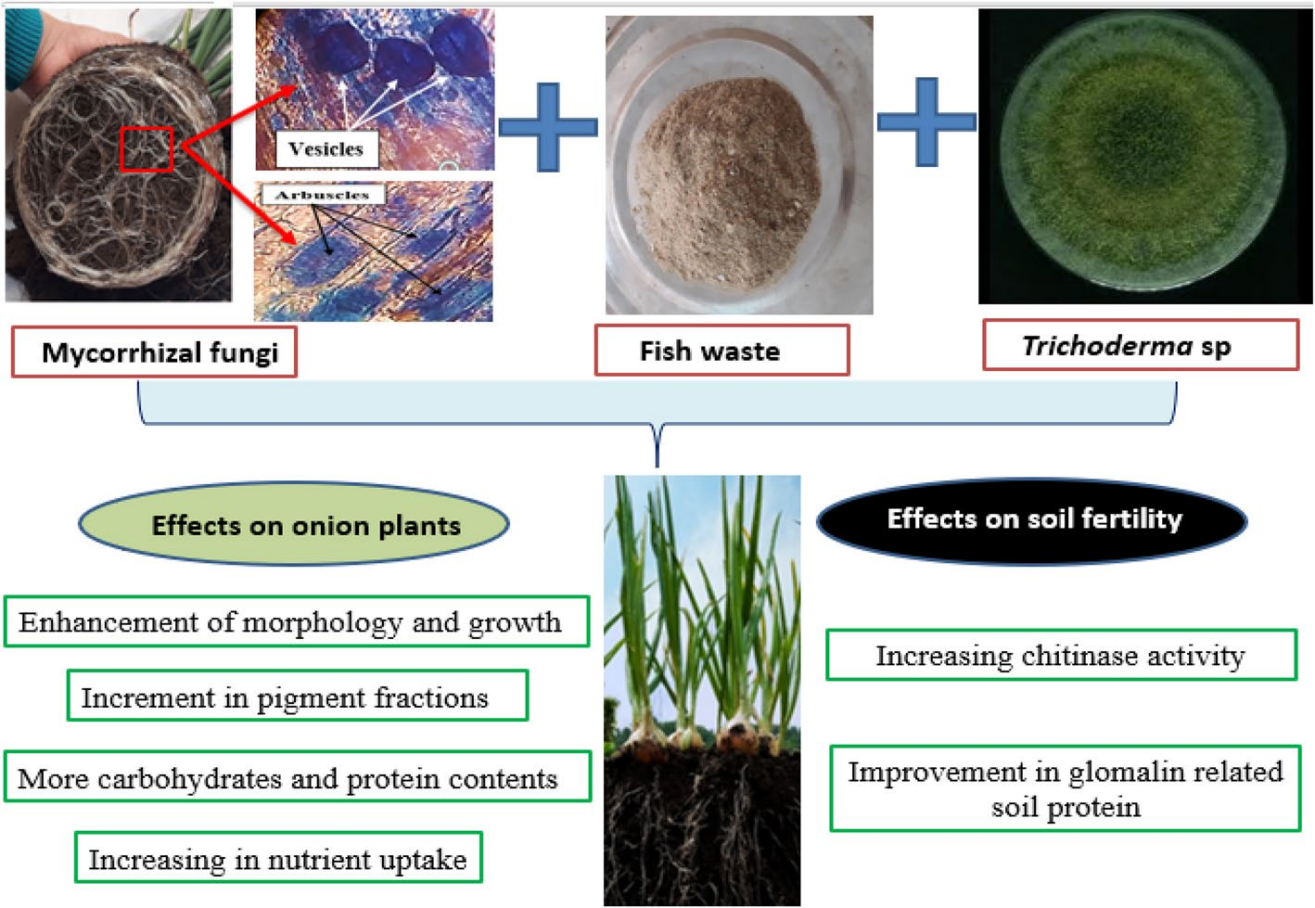
Model illustrating the effects of triple combination between AM fungi, *Trichoderma* sp. inoculations and FW application on onion plants and soil fertility

Apart from the macronutrients, the trend of the increase was repeated with micronutrients (Zn and Fe), where their contents were significantly increased (*p* < 0.05) in onion shoots and roots with AM fungi, *Trichoderma* sp. Besides, with FW additions, further augmentation in their contents was observed (Fig. 7) compared to their individual inoculations. The increased Zn content in onion roots and shoots was in harmony with Colla et al. [79] in lettuce and tomato co-inoculated with *Glomus intraradices* and *T. atroviride*. Moreover, Bagheri et al. [80] reported that AM fungal association exhibited high levels of P, K, Zn, and Mn in *Pistachio* plants. Therefore, in the nutrient-deficient soils, the incorporation of AM and *Trichoderma* sp. along with FW additions are of great significance for nutrient uptake and enhancing crop performance which may be attributed to improved plant nutritional status (N, P, K, Mg, Ca and Zn) via an increase in root surface.

## Conclusion

The suitable management of organic wastes by converting them to valuable resources is of utmost importance. Our results showed a good efficacy of FW in enhancing all agronomic growth parameters of onion plants grown in pots with the aid of AM fungi and *Trichoderma* sp. bio-inoculants. Moreover, leaf chlorophyll content, carotenoids, roots protein, total soluble carbohydrates and minerals nutrient content of onion plant improved with FW. Also, AM and *Trichoderma* sp. application along with FW amendments improved soil quality (glomalin related soil protein) and increased chitinase enzyme activity in the soil. Owing to their benefits on soil fertility and chitinase enzyme, FW as a compost progresses the condition of agricultural soils and increases growth and other physiological characteristics of onion plants. It can be concluded that FW is promising for enhancing the growth, development and quality of onion under potted conditions and further future research should evaluate these efficiencies strategies under field conditions.

## Methods

### Fish waste (Raw material)

Scales, heads, and fins of tilapia fish as well as shrimp peels were previously gathered from local fish markets close to Zagazig University, Egypt, and were used as the study's experimental materials (FW). These wastes were cleaned before being detergent-washed. These cleaned wastes were dried, crushed, sieved into fine powder, and used.

### Fungal inoculants isolation and preparation:

#### Isolation and identification of *Trichoderma* sp

*Trichoderma* sp. was isolated using a serial dilution plate approach from the rhizosphere of egg and pepper plants cultivated in the Egyptian soil of El-Sharkia Governorate. Under aseptic circumstances, one mL of suspension from each dilution was placed into sterile Petri plates containing Rose Bengal Medium [81]. After 3 days, *Trichoderma* colonies were picked and cultivated on potato dextrose agar (PDA) medium. By inoculating the fungal colonies and incubating them at 28 °C for 7 days, pure isolates were obtained. The morphological description of the *Trichoderma* isolate was verified by comparing macroscopic features on agar plates with those of a standard fungus atlas manual, as well as microscopic characteristics such as macroconidia, microconidia, as well as conidia shape and hyphae arrangement.

### Molecular identification of isolated fungus

The pure isolate of *Trichoderma* sp. was sub-cultured on a PDA medium and grown for 5 days at 30 °C and its genomic DNA was isolated using the CTAB technique [82, 83]. In the presence of liquid N_2_, grinding broke down the cell walls of fungal mycelia. The combination was then incubated at 65 °C with the addition of the CTAB extraction buffer before being purified using phenol, chloroform, and isoamyl alcohol (25:24:1). The genomic DNA was precipitated with cold isopropanol and thrice rinsed with cold 70% ethyl alcohol. Finally, 50 μL of sterile distilled water were used to dissolve the DNA.

### PCR Amplification and phylogenetic analysis

Using the ITS1 and ITS4 primers, The ITS1 and ITS2 and the inverting 5.8S coding rDNA were amplified [81]. In a total volume of 50 µL, each PCR reaction mixture comprised 5–10 ng genomic DNA, 1 µM of each ITS1/ITS4 primers, 5 µL of a 10X reaction buffer (50 mM KCl, 50 mM Tris–HCl; pH 8.3, 0.1 mg/mL bovine serum albumin (BSA), 3 mM MgCl_2_, 200 µM each of dNTP, and 2.5 U of Taq DNA polymerase (Promega, Mannheim, Germany). The sequences of the ITS1 and ITS4 primers are; 5 ‘-TCCGTAGGTGAACCTGCGG-3 ‘and 5′ TCCTCCGCTTATTGATAT GC-3′, respectively. The PCR technique includes 35 cycles of denaturation at 95 °C for 30 s, annealing at 56 °C for 30 s, and elongation at 72 °C for 1 min. Before DNA sequencing, the PCR amplicon was resolved using a 8% agarose gel and purified using a specific PCR purification kit (Accu Prep® PCR DNA Purification Kit, K-3034–1, Bioneer Corporation, South Korea). MacrogenInc, (South Korea) sequenced the purified PCR products. All inter transcribed spacer sequencing work was also performed by MacrogenInc, (South Korea) and was carried out on both strands of the submitted DNA fragments. The purified PCR products were sequenced using an ABI 377 DNA. Auto sequencer (PerkinElmer, Applied Biosystems Div., Waltham, USA) based on the same primers mentioned before.

### Isolation and identification of AM fungi

Spores of AM fungi were isolated from rhizospheric soil of El-Sharkia Governorate, Egypt via wet sieving and decanting technique [84]. The spores were then separated and mounted using polyvinyl lactoglycerol alcohol. Approximately 200 g of individual air-dried soil was distributed in 2 L of water in a large jar and the suspension was left intact for 10–15 min. The suspension was then decanted through the stack of sieves 400, 250, 180 and 38 μm (arranged in decreasing order of mesh size from top to bottom), this process was repeated 2–3 times and the residue from each sieve was collected into a small flask with the aid of glass funnel with a little distilled water. The morphology of spores and sporocarps of AM fungi were observed and then their characters were identified by using Manual for identification [85]. The mixture of identified AM fungal spores of *Funneliformis mosseae, F. constrictum, Gigaspora margarita* and *Rhizophagus irregularis* together in pots filled with sterilized sandy clay soil were propagated with Sudan grass (*Sorghum sudanenses* Pers.) roots as an appropriate trap plant for two cycles of 5 months in the greenhouse.

### FW application under the greenhouse conditions

The soil utilized in this study was gathered from a field where onions were formerly grown in El-Sharkia Governorate. After sterilizing the soil with 2% formaldehyde, it was left unattended for 25 days for leakage of volatile poisonous substances. *Allium cepa* L. (Golden onion, Giza 20) seeds were obtained from the Agricultural Research Center in Giza, Egypt. They were surface disinfected with 2% sodium hypochlorite for 10 min, washed with sterile distilled water afterward, and then propagated in a nursery greenhouse for seedling production and utilized as the test plant. After receiving permission from the Agricultural Research Center in Giza, Egypt, onion seeds were acquired.

The soil samples of 2 kg were put into sterilized plastic pots (25 cm diam.) and then used for the growth of onion seedlings (2 seedlings/ pot) after transplanting (After 50 days of sowing) in the greenhouse. One hundred g/pot enclosing AM fungal colonized Sudan grass roots and soil mixture enclosed spores (AM fungal inocula) were added during transplanting. *Trichoderma* sp. inoculum was applied via the addition of 25 mL of spore suspension (2 × 10^7^conidia/ml) / pot in the onion rhizosphere. The control pot did not receive any fungal inoculum but only received 25 mL sterilized distilled water and 100 g sterilized soil. Also, FW of 0.75% (w/w) concentration was added during the transplanting of surrounding onion seedlings. As a consequence, the following 8 different treatments were administered to the potted plants in 5 replicates following the design described above:

- Treatment 1- (Control): Onion seedlings only.
- Treatment 2- (FW): Onion seedlings amended with FW.
- Treatment 3– (AM): Onion seedlings inoculated with AM fungi.
- Treatment 4- (Trichoderma sp.): Onion seedlings inoculated with Trichoderma sp.
- Treatment 5- (AM + Trichoderma sp.): Onion seedlings inoculated with AM fungi and Trichoderma sp.
- Treatment 6- (Trichoderma sp. + FW): Onion seedlings inoculated with Trichoderma sp. and amended with FW.
- Treatment 7 – (AM + FW): FW amendment and AM fungal inoculation of onion seedlings.
- Treatment 8 – (AM + Trichoderma sp. + FW): Onion seedlings inoculated with Trichoderma sp. and AM fungi and amended with FW.

### Plant and soil sampling

Plant samples from all treatments were collected for morphological and physiological assessment 8 weeks after transplanting and applying FW. Besides, samples of rhizospheric soil were taken for glomalin and chitinase enzyme activity.

### Determination of AM fungal colonization percentage

AM fungal colonized plant roots from each treatment were washed separately with tap water, cut into small pieces of 0.5–1 cm in length then cleared with 10% KOH solution. The root colonization percentage was evaluated by staining with 0.05% trypan blue for 15 min at 90 °C as designated by Phillips and Hayman [86]. Afterwards, the stained roots were detected with an optical microscope and the percentage of root colonization (F %) was determined according to the following equation:

$$\mathrm{\%\;AM\;fungal\;Colonization}= \frac{(\mathrm{ The\;total\;number\;of\;AM\;colonized\;segments})}{(\mathrm{ Total\; number\;of\;root\;segments\;observed })} \times 100$$

### Morphological assessments

#### Root length and plant height

Onion roots were carefully washed under tap water to remove soil particles and their lengths were obtained as the mean value of the longest root of each plant from the bulb base. Also, plant height was measured as the topmost height of the main leaf to the onion base bulb. The data was recorded as the average height or length in cm per plant (cm/plant).

#### Number of leaves

Total leaves of *Allium* plants were counted from each pot. The reading was recorded as the average number of all leaves of each plant of all pots divided by the number of total plants for each treatment.

#### Onion leaf area

The leaf area (cm^2^) of onion leaves was recorded [87] according to the following equation:

$$Leaf\;area\;(cm2) = Lcm\;x\;Wcm\;x\;0.785$$

where L is the length of the leaf and W is the maximum width of the leaf.

#### Neck and bulb diameter of onion

The neck diameter of the onion plants after 8 weeks of transplanting and FW application was measured at 3 cm above the surface of the soil and the bulb diameter was recorded after uprooting the plant using a thread.

#### Total fresh and dry weights of shoots and roots

After taking the fresh weight (Fwt), the shoot and root plant samples were dried in an electric oven at 60 °C till constant weight. The total fresh weight (TFwt) and total dry weight (TDwt) of onion shoots and roots were measured and recorded in grams per plant (g/plant). Total shoot weight represents the weight of both onion leaves and bulb.

### Physiological assessments

#### Estimation of photosynthetic pigments content

The Fwt of onion leaves (0.25 g) from all treatments was crushed separately in a mortar using 10 mL of 85% aqueous acetone solution. The homogenate was centrifuged and the absorbance was measured at 663, 644, 452.5 nm using a UV–visible Spectrophotometer, RIGOL (Model Ultra-3660). The following three equations were applied to estimate the content of the pigments of samples in terms of µg/mL according to Metzner et al. [88].

$$Chl.a\hspace{0.17em}=\hspace{0.17em}10.3 A663 - 0.918 A644$$

$$Chl.a\hspace{0.17em}=\hspace{0.17em}10.3 A663 - 0.918 A644$$

$$Carotenoid = 4.2 A452.5 - (0.0264 Chl.a + 0.426 Chl.b)$$

The calculated pigment content was then expressed as mg/g Fwt as follows:

$$\mathrm{Chl}.\mathrm{a}=\frac{\mathrm{Chl}.\mathrm{a }\left(\frac{\mathrm{\mu g}}{\mathrm{mL}}\right)\mathrm{ X\;Extract\; volume }\;(\mathrm{mL}) }{\mathrm{Fwt\;of\;sample }\left(\mathrm{g}\right)\;\mathrm{X }\;1000}$$

#### Estimation of total soluble carbohydrates content

Carbohydrates in onion leaves after FW and fungal application were estimated by the phenol sulphuric acid method as described by Dubois et al. [89] after extracting 0.1 g of dried leaves with 2.5 N HCl. The absorbance was read at 490 nm and carbohydrates concentration was calculated in terms of mg/mL using a glucose standard curve and its content was then expressed as mg/g Dwt. as follows:

$$\mathrm{mg}/\mathrm g\;\mathrm{glucose}\;=\frac{\mu\mathrm g/\mathrm{mL}\times\;\mathrm{Extract}\;\mathrm{volume}\times\;\mathrm{Dilution}\;\mathrm{fa}c\mathrm{tor}}{\mathrm{Dry}\;\mathrm{weight}\;\mathrm{of}\;\mathrm{sample}\;\left(\mathrm g\right)\;\times\;1000}$$

#### Assessment of total soluble protein contents in onion roots

Using BSA as standard, the total soluble protein content in onion roots was calculated as mg/g Fwt after separate homogenization of a known onion root Fwt for all treatments (0.5 g) in NaOH (1 N) as well as centrifugation [90]. One mL of solubilized protein was combined with newly prepared alkaline copper solution for 10 min. Folin–Ciocalteau reagent was then added and left for 30 min. Afterwards, a UV–visible Spectrophotometer measured the optical density at 700 nm.

#### Estimation of chitinase activity

### Colloidal chitin preparation

Colloidal chitin was prepared according to the technique of Reid and Ogrydiziak [91]. Ten grams of milled chitin were suspended in 100 mL of H_3_PO_4_ (85%) and stored at 4 °C for 24 h, this mixture was suspended in 1 L of deionized water and washed until the pH was 5. Sodium hydroxide (NaOH) (1 N) was then added to adjust the pH to 7, afterwards centrifuged at 4000 rpm for 20 min. The pelleted chitin was stored at 4 °C. Chitin concentration was calculated by drying to constant weight [92].

### Chitinase activity

Chitinase activity was measured in 10 g air dried rhizosphere in a 60 mL glass bottle. The soil was treated with 1.5 mL toluene for 15 min, followed by the addition of 10 mL of 1% (w/v) colloidal chitin. The mixture was shaken and incubated at 37 °C for 18 h. After incubation, 10 mL of water was added to each bottle and 10 mL of the resulting suspension was centrifuged for 20 min at 4000 g. One mL of the clear supernatant was used to determine the amount of N-acetylglucosamine (NAG) released, following the method of Mian et al. [93]. One mL of the clear supernatant was incubated 1 mL of 1% of dinitrosalicylic acid (DNS) for 5 min in a boiling water bath. The NAG production was evaluated by obtaining spectrophotometer absorbance at 535 nm. Chitinase activity was expressed as μmoles of NAG released per hour per gram of soil.

#### GRSP analyses

As reported by Wright and Upadhyaya [72], the concentrations of easily extractable glomalin related soil protein (EE-GRSP) and total glomalin related soil protein (T-GRSP) have been estimated. In general, T-GRSP was extracted from 1 g of air-dried onion soil with 8 mL of 50 mmol / L citrate solution (pH 7.0) and then autoclaved for 60 min at 121 °C. After centrifugation at 6000 rpm for 10 min, the supernatant was separated. This step was performed several times on the same sample until there was no red-brown color in the solution. The supernatant was collected and stored at 4 °C. EE-GRSP was extracted with 8 mL of 20 mmol/L citrate solution (pH 7) from 1 g of air-dried onion soil sample and then autoclaved (at 121 °C for 30 min). Centrifuging was performed for 10 min at 6000 rpm, and the supernatant was collected and kept at 4 °C. Bradford analysis at 590 nm and BSA as a standard were used to assess T-GRSP or EE-GRSP in the supernatant [94].

### Minerals analysis

For assessment of onion minerals nutrient contents, shoots and roots of all treatments were dried separately, sieved through a 0.5 mm sieve and digested with a mixture of sulphuric acid: hydrogen peroxide at the ratio of 2:1 (v/v) [95]. Afterwards, the calcium (Ca), potassium (K), magnesium (Mg), zinc (Zn) and iron (Fe) contents were determined spectrophotometrically in the digested samples in the Faculty of Agriculture Central lab, Zagazig University by using Inductively Coupled Plasma Spectrometry (Ultima 2 JY Plasma). Total phosphorus (P) of shoot and root was determined at 620 nm [96]. Moreover, total nitrogen (N) of shoot and root dried samples was measured using the Kjeldahl method [97] after digestion with concentrated sulfuric acid (98.8%) and digestion mixture.

### Statistical analysis

Using SPSS software version 15 (SPSS, Richmond VA, USA), the results were expressed as the mean of 5 replicates ± standard deviation (SD), and the means were compared for statistical significance by one way ANOVA (significance level (*p* < 0.05), the confidence interval was 95%, before applying post hoc procedure. Additionally, three-way ANOVA was applied to scrutinize the effects of AM fungi, *Trichoderma* sp. inoculation, FW addition and their interactions on the total biomass, chlorophyll, carbohydrates and chitinase enzyme activities. The Origin 2017 programme for graphing and data analysis was used to create the figures.

## Supplementary Information

**Additional file 1: Figure S1.** The phylogenetic relationship between *Trichoderma *sp. (*Trichoderma viride* strain RA1, ON479613) showing the ITS sequences of closely related fungal strains retrieved from NCBI GenBank. The percentages of replicate trees in which the associated taxa clustered together in the bootstrap test (1000 replicates) are shown next to the branches. The tree is drawn to scale, with branch lengths in the same units as those of the evolutionary distances used to infer the phylogenetic tree. The evolutionary distances were computed using the maximum composite likelihood method and are in the units of the number of base substitutions per site. Evolutionary analyses were conducted in MEGA7.

## Data Availability

The [GenBank NCBI] contains the datasets that were created and analyzed for the current investigation https://www.ncbi.nlm.nih.gov/nuccore/ON479613.1/.

## Abbreviations

AM: Arbuscular Mycorrhiza
ANOVA: Analysis of variance
BSA: Bovine serum albumin
FW: Fish waste
GRSP: Glomalin-related soil protein
SD: Standard deviation
TDwt: Total dry weight
TFwt: Total fresh weight

## References

[1] Ranaweera SRM, Nanayakkara CM, Tennakoon NA (2010) Comparison of the effects of organic fertilizers with inorganic fertilizers on the growth of eight months old coconut seedlings and the nutrient availability and soil microbial activity of soils. Coconut Research Institute, Sri Lanka. Proceedings of the 15th International Forestry and Environment Symposium, pp 93 -100.

[2] Savci S (2012). An Agricultural pollutant: Chemical fertilizer. International Journal of Environmental Science and Development.

[3] Ranasinghe A, Jayasekera R, Kannangara S, Rathnayake S (2019). Effect of nutrient enriched organic liquid fertilizers on growth of *Albemonchus esculentus*. Journal of Environment Protection and Sustainable Development.

[4] Fernandez V, Brown PH (2013). From plant surface to plant metabolism: the uncertain fate of foliar–applied nutrients. Frontiers in Plant Sciences Plant Nutrition.

[5] Metwally RA, Soliman SA, Abdel Latef AA, Abdelhameed RE (2021). The individual and interactive role of arbuscular mycorrhizal fungi and *Trichoderma viride* on growth, protein content, amino acids fractionation, and phosphatases enzyme activities of onion plants amended with fish waste. Ecotoxicol Environ Saf.

[6] Abdel-Fattah GM, Mohamedin AH (2000). Interactions between a vesicular-arbuscular mycorrhizal fungus (*Glomus intraradices*) and *Streptomyces coelicolor* and their effects on sorghum plants grown in soil amended with chitin of brawn scales. Biol Fertil Soils.

[7] Gadelrab HM, Elamin SM (2013) Effect of different organic fertilizers on growth, yield and total soluble solid of the onion (*Allium cepa* L.) variety Baftaim-s. Int. J. Agric. Vet. Sci. 14 (1), 61.

[8] Petrovic B, Pokluda R (2020). Influence of organic fertilizers on onion quality. Pol J Environ Stud.

[9] Vosátka M, Látr A, Albechtova J (2015). Bioadditives for vegetables growth optimization in protected cultivation. Acta Hortic.

[10] Ismail A, Riaz M, Akhtar S, Ismail T, Amir M, Zafarul-Hye M (2014). Heavy metals in vegetables and respective soils irrigated by canal, municipal waste and tube well water. Food Addit Contam Part B.

[11] Al-Zabee M, Al-Maliki S (2019). Interactions between biofertilizers and chemical Fertilizers affected soil biological properties and potato yield. Euphrates J Agric Sci.

[12] Medina A, Azcón R (2010). Effectiveness of the application of arbuscular mycorrhiza fungi and organic amendments to improve soil quality and plant performance under stress conditions. J soil sci plant nutr.

[13] Nurbaity A, Uratel GC, Hamdani JS (2019). Mycorrhiza Enhanced Protein and Lipid Contents of Potatoes Grown on Inceptisol with Addition of Organic Matter. J Trop Soils.

[14] Medina A, Vassilev N, Alguacil MM, Roldán A, Azcón R (2004). Increased plant growth, nutrient uptake, and soil enzymatic activities in a desertified mediterranean soil amended with treated residues and inoculated with native mycorrhizal fungi and a plant growth promoting yeast. Soil Sci.

[15] Ellyzatul AB, Nornasuha Y, Nashriyah M, Khandaker MM (2018). Effects of fish waste effluent on the growth, yield and quality of *Cucumis sativus* L. J Agrobiotech.

[16] Jayasundara JMNP, Jayasekara R, Ratnayake RMCS (2016) Liquid organic fertilizers for growth enhancement of *Abelmoschus esculentus* (L.) Moench and *Alternanthera sessilis* (L.) DC. Tropical Plant Research 3 (2): 334–340.

[17] Arumugam G, Balraj TH, Palani S (2014). A liquid fermented fish waste on the growth characteristics of *Solanum melongena*. J Chem Pharm Res.

[18] Suryawanshi N, Jujjavarapu SE, Ayothiraman S (2019). Marine shell industrial wastes–an abundant source of chitin and its derivatives: constituents, pretreatment, fermentation, and pleiotropic applications-a revisit. Int J Environ Sci Technol.

[19] Sharp RG (2013). A Review of the applications of chitin and its derivatives in agriculture to modify plant-microbial interactions and improve crop yields. Agronomy.

[20] Nandhini E, Raja S, Logankumar K, Lekshmanaswamy M, Abirami P (2014) Conversion of fish wastes into liquid fertilizer by using microbes for sustainable organic Agriculture. Conference Paper.

[21] Liu CH, Liu Y, Fan C (2013). Kuang SZ (2013) The effects of composted pineapple residue return on soil properties and the growth and yield of pineapple. J Soil Sci Plant Nutr.

[22] Ahmad M, Pataczek L, Hilger TH, Zahir ZA, Hussain A, Rasche F, Schafleitner R, Solberg SØ (2018). Perspectives of microbial inoculation for sustainable development and environmental management. Front Microbiol.

[23] Abdelhameed RE, Metwally RA (2019). Alleviation of cadmium stress by arbuscular mycorrhizal symbiosis. Int J Phytoremediation.

[24] Evelin H, Devi TS, Gupta S, Kapoor R (2019). Mitigation of salinity stress in plants by arbuscular mycorrhizal symbiosis: Current Understanding and New Challenges. Front Plant Sci.

[25] Abdelhameed RE, Abu-Elsaad NI, Abdel Latef AAH, Metwally RA (2021) Tracking of Zinc Ferrite Nanoparticle effects on pea (*Pisum sativum* L.) plant growth, pigments, mineral content and arbuscular mycorrhizal colonization. Plants, 10, 583. 10.3390/plants10030583 PMC8003511 33808615

[26] Smith SE, Read DJ (2008). Mycorrhizal Symbiosis.

[27] Metwally RA, Abdelhameed RE (2019). Impact of Ridomil, Bavistin and Agrothoate on arbuscular mycorrhizal fungal colonization, biochemical changes and potassium content of cucumber plants. Ecotoxicology.

[28] Metwally RA (2020) Arbuscular mycorrhizal fungi and *Trichoderma viride* cooperative effect on biochemical, mineral content, and protein pattern of onion plants. J Basic Microbiol. 1–10. 10.1002/jobm.202000087 32367554

[29] Harman GE, Herrera-Estrella AH, Horwitz BA, Lorito M (2012). Trichoderma – from basic biology to biotechnology. Microbiology.

[30] Metwally RA, Al-Amri SM (2019). Individual and interactive role of *Trichoderma viride* and arbuscular mycorrhizal fungi on growth and pigment content of onion plants. Lett Appl Microbiol.

[31] Metwally RA, Abdelhameed RE, Soliman SA, Al-Badwy AH (2022). Potential use of beneficial fungal microorganisms and C-phycocyanin extract for enhancing seed germination, seedling growth and biochemical traits of *Solanum lycopersicum* L. BMC Microbiol.

[32] Brzezinska MS, Jankiewicz U, Burkowska A, Walcza M (2014). Chitinolytic microorganisms and their possible application in environmental protection. Curr Microbiol.

[33] Rattanakit N, Plikomol A, Yano S, Wakayama M, Takashi T (2002). Utilization of shrimp shellfish waste as a substrate for solid-state cultivation of *Aspergillus* sp. Evaluation of a culture based on chitinase formation which is necessary for chitin assimilation. Biosci Bioeng.

[34] Agrawal T, Kotasthane AS (2012). Chitinolytic assay of indigenous Trichoderma isolates collected from different geographical locations of Chhattisgarh in Central India. Springerplus.

[35] Loc NH, Huy ND, Quang HT, Lan TT, Thu Ha TT (2019). Characterisation and antifungal activity of extracellular chitinase from a biocontrol fungus, *Trichoderma asperellum* PQ34. Mycology.

[36] Mejía C, Ardila HD, Espinel C, Brandão PFB, Villamizar L (2021) Use of *Trichoderma koningiopsis* chitinase to enhance the insecticidal activity of *Beauveria bassiana* against *Diatraea saccharalis*. *Interactions with the environment*. Journal of Basic Microbiology. 61( 9): 814–824. 10.1002/jobm.202100161 34312885

[37] Pozo MJ, Azcon-Aguilar C, Dumas-Gaudot E, Barea JM (1998). Chitosanase and chitinase activities in tomato roots during interactions with arbuscular mycorrhizal fungi or Phytopthorparasitica. J Exp Bot.

[38] Rillig MC, Mummey DL (2006). Mycorrhizas and soil structure. New Phytol.

[39] Cheng H, Giri B, Wu Q, Zou Y, Kuča K (2021). Arbuscular mycorrhizal fungi mitigate drought stress in citrus by modulating root microenvironment. Archives of Agronomy and Soil Science.

[40] Wu QS, Cao MQ, Zou YN, He XH (2014). Direct and indirect effects of glomalin, mycorrhizal hyphae, and roots on aggregate stability in rhizosphere of trifoliate orange. Sci Rep.

[41] Yang Y, He C, Huang L, Ban Y, Tang M (2017). The effects of arbuscular mycorrhizal fungi on glomalin-related soil protein distribution, aggregate stability and their relationships with soil properties at different soil depths in lead-zinc contaminated area. PLoS ONE.

[42] Li H, Liu G, Gu J, Chen H, Shi H, Abd Elbasit MAM, Hu F (2021). Response of soil aggregate disintegration to the different content of organic carbon and its fractions during splash erosion. Hydrol Process.

[43] Cai F, Druzhinina IS (2021). In honor of John Bissett: authoritative guidelines on molecular identification of Trichoderma. Fungal Diversity.

[44] Druzhinina IS, Kopchinskiy AG, Komon M, Bissett J, Szakacs G, Kubicek CP (2005). An oligonucleotide barcode for species identification in *Trichoderma* and *Hypocrea*. Fungal Genet Biol.

[45] Tamura K, Nei M, Kumar S (2004). Prospects for inferring very large phylogenies by using the neighbor-joining method. Proc Natl Acad Sci USA.

[46] Sutton JC (1973). Development of vesicular-arbuscular mycorrhiza in crop plants. Can J Bot.

[47] Johansson J, Paul LR, Finlay RD (2004). Microbial interactions in the mycorrhizosphere and their significance for sustainable agriculture. FEMS Microbiol Ecol.

[48] Júnior AGG, Pereira RA, Sodre´ GA, do Sacramneto CK, Gross E, (2018). Inoculation with arbuscular micorrizhal fungi and organic compost from cocoa shell positively influence the growth and mineral nutrition of soursop plants (*Annona muricata* L.). Rev Bras Frutic.

[49] Ramos AC, Martins MA (2010) Fisiologia de micorrizas arbusculares. In: Siqueira JO, Souza FA, Cardoso EJBN, Tsai SM (Eds.), Micorrizas: 30 Anos de Pesquisa no Brasil. Editora UFLA, Lavras.

[50] Sousa C, Menezes RS, Sampaio EV, Oehl F, Maia LC, Garrido M, Lima F (2012). Occurrence of arbuscular mycorrhizal fungi after organic fertilization in maize, cowpea and cotton intercropping systems. Acta Sci Agron.

[51] Fracchia S, Garcia-Romera I, Godeas A, Ocampo JA (2000). Effect of the saprophytic fungus *Fusarium oxysporum* on arbuscular mycorrhizal colonization and growth of plants in greenhouse and field trials. Plant Soil.

[52] Nieto-Jacobo MF, Steyaert JM, Salazar-Badillo FB, Nguyen DV, Rostas M, Braithwaite M, De Souza JT, Jimenez-Bremont JF (2017). Environmental growth conditions of Trichoderma spp. affects indole acetic acid derivatives, volatile organic compounds, and plant growth promotion. Front Plant Sci.

[53] Radziemska M, Vaverková MD, Adamcová D, Brtnický M, Mazur Z (2018). Valorization of fish waste compost as a fertilizer for agricultural use. WASTE BIOMASS VALORI.

[54] Srinivasan R, Jeevan Rao K, Reza SK, Padua S, Dinesh D (2016). Dharumarajan S (2016) Influence of inorganic fertilizers and organic amendments on plant nutrients and soil enzyme activities under incubation. International Journal of Bio-resource and Stress Management.

[55] Abdelhameed RE, Metwalley RA (2018). Mitigation of salt stress by dual application of arbuscular mycorrhizal fungi and salicylic acid. Agrochimica.

[56] Metwally RA, Abdelhameed RE (2018). Synergistic effect of arbuscular mycorrhizal fungi in growth and physiology of salt-stressed *Trigonella foenum-graecum* plants. Biocatal Agric Biotechnol.

[57] Begum N, Qin C, Ahanger MA, Raza S, Khan MI, Ashraf M, Zhang AN, L,  (2019). Role of arbuscular mycorrhizal fungi in plant growth regulation: Implications in abiotic stress tolerance. Front Plant Sci.

[58] Dalorima T, Khandaker MM, Zakaria AJ, Hasbullah M (2018). Impact of organic fertilizations in improving BRIS soil conditions and growth of watermelon (*Citrullus Lanatus*). Bulgarian J Agr Sci.

[59] Khandaker MM, Rohani F, Dalorima T, Mat N (2017) Effects of different organic fertilizers on growth, yield and quality of *Capsicum annuum* L. Var. Kulai (Red ChilliKulai). Biosci., Biotech. Res. Asia, 14(1): 185–192.

[60] Cruz R, Baptista P, Cunha S, Pereira AJ, Casal S (2012). Carotenoids of Lettuce (*Lactuca sativa* L.) Grown on Soil Enriched with Spent Coffee Grounds. Molecules.

[61] Badar R, Qureshi SA (2012). Use of *Trichoderma hamatum* alone and in combination with rhizobial isolates as biofertilzer for improving the growth and strength of sunflower. J Basic Appl Sci Res.

[62] Bougnom BP, Knapp BA, Elhottová D, Koubová A, Etoa FX, Insam H (2010). Designer compost with biomass ashes for ameliorating acid tropical soils, Effects on the soil microbiota. Appl Soil Ecol.

[63] Sheng M, Tang M, Zhang F, Huang Y (2011). Influence of arbuscular mycorrhiza on organic solutes in maize leaves under salt stress. Mycorrhiza.

[64] Metwally RA, Azab HS, Al-Shannaf HM, Rabie GH (2022) Prospective of mycorrhiza and *Beauvaria bassiana* silica nanoparticles on *Gossypium hirsutum* L. plants as biocontrol agent against cotton leafworm, *Spodoptera littoralis*. BMC Plant Biol 22, 409. 10.1186/s12870-022-03763-x PMC9392270 35987628

[65] Shahsavani SH, Abaspour A, Parsaeian M, Unesi Z (2017). Effect of fish waste, chemical fertilizer and biofertilizer on yield and yield components of bean (*Vigna Sinensis*) and some soil properties. Iranian journal of pulses research.

[66] Gong X, Fu Y, Jiang D, Li G, Yi X, Peng Y (2007). L-Arginine is essential for conidiation in the filamentous fungus *Coniothyrium minitans*. Fungal Gen Biol.

[67] Paul T, Halder SK, Das A (2015) Production of chitin and bioactive materials from Black tiger shrimp (*Penaeus monodon*) shell waste by the treatment of bacterial protease cocktail. *3 Biotech* 5, 483–493. 10.1007/s13205-014-0245-6 PMC4522719 28324551

[68] Brzezinska MS, Lalke-Porczyk E, Donderski W (2008). Occurrence and activity of microorganisms in shrimp waste. Curr Microbiol.

[69] Lanfranco L, Garnero L, Bonfante P (1999). Chitin synthase genes in the arbuscular mycorrhizal fungus *Glomus versiforme*: full sequence of a gene encoding a class IV chitin synthase. FEMS Microb Lett.

[70] Haran S, Schickler H, Oppenheim A, Chet I (1995). New components of the chitinolytic system of *Trichoderma harzianum*. Nucleic Acid Res.

[71] Sharma V, Shanmugam V (2011). Purification and characterization of an extracellular 24 kDa chitobiosidase from the mycoparasitic fungus *Trichoderma saturnisporum*. J Basic Microbiol.

[72] Wright SF, Upadhyaya A (1998). A survey of soils for aggregate stability and glomalin, a glycoprotein produced by hyphae of arbuscular mycorrhizal fungi. Plant Soil.

[73] Gryndler M, Jansa J, Hrˇselová H, Chvátalová I, Vosátka M (2003). Chitin stimulates development and sporulation of arbuscular mycorrhizal fungi. Appl Soil Ecol.

[74] Driver JD, Holben WE, Rillig MC (2005). Characterization of glomalin as a hyphal wall component of arbuscular mycorrhizal fungi. Soil Biol Biochem.

[75] Meng L-L, He J-D, Zou Y-N, Wu Q-S, Kuča K (2020). Mycorrhiza-released glomalin-related soil protein fractions contribute to soil total nitrogen in trifoliate orange. Plant Soil Environ.

[76] Luginbuehl LH, Menard GN, Kurup S, Van Erp H, Radhakrishnan GV, Breakspear A (2017). Fatty acids in arbuscular mycorrhizal fungi are synthesized by the host plant. Science.

[77] Mitra D, Navendra U, Panneerselvam U, Ansuman S, Ganeshamurthy AN, Divya J (2019) Role of mycorrhiza and its associated bacteria on plant growth promotion and nutrient management in sustainable agriculture. Int. J.LSAS

[78] Li RX, Cai F, Pang G, Shen QR, Li R, Chen W (2015). Solubilisation of phosphate and micronutrients by *Trichoderma harzianum* and its relationship with the promotion of tomato plant growth. PLoS ONE.

[79] Colla G, Rouphael Y, Mattia ED, El-Nakhel C, Cardarelli M (2014). Co-inoculation of *Glomus intraradices* and *Trichoderma atroviride* acts as a biostimulant to promote growth, yield and nutrient uptake of vegetable crops. J Sci Food Agric.

[80] Bagheri V, Shamshiri MH, Shirani H, Roosta H (2012). Nutrient uptake and distribution in mycorrhizal pistachio seedlings under drought stress. J Agric Sci Technol.

[81] Zhou C, Guo RT, Ji SD, Fan HJ, Wang JJ, Wang YC, Liu ZH (2020). Isolation of *Trichoderma* from forestry model base and the antifungal properties of isolate TpsT17 toward *Fusarium oxysporum*. Microbiol Res.

[82] Wu ZH, Wang TH, Huang W, Qu YB (2001). A simplified method for chromosome DNA preparation from filamentous Fungi. Mycosystema.

[83] White TJ, Bruns T, Lee S, Taylor J, Innis MA, Gelfand DH, Sninsky JJ, White TJ (1990). Amplification and direct sequencing of fungal ribosomal RNA genes for phylogenetics. PCR Protocols: a guide to methods and applications.

[84] Gerdemann JW, Nicolson TH (1963). Spores of mycorrhizal Endogone species extracted from soil by wet sieving and decanting. Trans Br Mycol Soc.

[85] Manimegalai V, Selvaraj T, Ambikapathy V (2011). Studies on isolation and identification of VAM fungi *in Solanum viarum* Dunal of medicinal plants. Adv Appl Sci Res.

[86] Phillips JM, Hayman DS (1970). Improved procedures for clearing roots and staining parasitic and vesicular-arbuscular mycorrhizal fungi for rapid assessment of infection. Trans Br Mycol Soc.

[87] Shih SF, Snyder GH (1984). Leaf area index and dry biomass of taro. American Society of Agronomy.

[88] Metzner H, Rau H, Senger H (1965). Untersuchungen Zur Synchronisierbarkeit einzelner Pigment-Mangel Mutanten Von Chlorella. Planta.

[89] Dubois M, Gilles KA, Hamilton JK, Rebers PA, Smith F (1956). Calorimetric method for determination of sugars and related substances. Anal Chem.

[90] Lowry OH, Rosbrough NJ, Farr AL, Randall RJ (1951). Protein measurement with the Folin phenol reagent. J Biol Chem.

[91] Reid JD, Ogrydiziak DM (1981). Chitinase–over producing mutant of *Serratia marcescens*. Appl and Environ Microbiol.

[92] El-Sayed ASA (2005) Studies on some thermophilic chitin degrading fungi. M. Sc Thesis. Faculty of science, Zagazig Univ., Egypt.

[93] Mian IH, Godoy G, Shelby RA, Rodriguez-Kabana R, Morgan-Jones G (1982). Chitin amendments for control of Meloidogyne arenaria in infested soil. Nematropica.

[94] Bradford MM (1976). A rapid and sensitive method for the quantification of microgram quantities of protein utilizing the principle of protein-dye binding. Annu Rev Biochem.

[95] Lowther JR (1980). Use of a single sulphuric acid- hydrogen peroxide digest for the analysis of *Pinus radiata* needles. Commun Soil Sci Plan.

[96] Murphy J, Riley J (1962). A modified single solution method for the determination of phosphate in natural waters. Anal Chim Acta.

[97] Nelson DW, Sommers LE (1973). Determination of total nitrogen in plant material. Agron J.

