# Supplementary material for: Assessment of beneficial fungal microorganism’s bio-efficacy in stimulating morphological and physiological parameters of Allium cepa plants grown in soil amended with fish wastes
Source: BMC Plant Biol. 2022 Dec 29;22:617. doi: 10.1186/s12870-022-03965-3 (PMC9798718; doi:10.1186/s12870-022-03965-3)
Supplement: Supplementary file 1 — Additional file 1: Figure S1. The phylogenetic relationship between Trichoderma sp. (Trichoderma viride strain RA1, ON479613) showing the ITS sequences of closely related fungal strains retrieved from NCBI GenBank. The percentages of replicate trees in which the associated taxa clustered together in the bootstrap test (1000 replicates) are shown next to the branches. The tree is drawn to scale, with branch lengths in the same units as those of the evolutionary distances used to infer the phylogenetic tree. The evolutionary distances were computed using the maximum composite likelihood method and are in the units of the number of base substitutions per site. Evolutionary analyses were conducted in MEGA7. [file 12870_2022_3965_MOESM1_ESM.docx]

**Figure S1:** The phylogenetic relationship between *Trichoderma* sp. (*Trichoderma viride* strain RA1, ON479613) showing the ITS sequences of closely related fungal strains retrieved from NCBI GenBank. The percentages of replicate trees in which the associated taxa clustered together in the bootstrap test (1000 replicates) are shown next to the branches. The tree is drawn to scale, with branch lengths in the same units as those of the evolutionary distances used to infer the phylogenetic tree. The evolutionary distances were computed using the maximum composite likelihood method and are in the units of the number of base substitutions per site. Evolutionary analyses were conducted in MEGA7.
